# Evolving guidelines in the use of topical nonsteroidal anti-inflammatory drugs in the treatment of osteoarthritis

**DOI:** 10.1186/1471-2474-15-27

**Published:** 2014-01-21

**Authors:** Casilda M Balmaceda

**Affiliations:** 1 NY Neurological Consultants, PC, 300 Fort Washington Avenue, Suite 1, New York 10032, New York

**Keywords:** Osteoarthritis, Pain, NSAIDs, Topical, Guidelines

## Abstract

**Background:**

Nonsteroidal anti-inflammatory drugs (NSAIDs) are a standard treatment for osteoarthritis (OA), but the use of oral NSAIDs has been linked to an elevated risk for cardiovascular and gastrointestinal adverse events and renal toxicity. Topical NSAIDs are thought to afford efficacy that is comparable to oral formulations while reducing widespread systemic drug exposure, which may provide a benefit in terms of safety and tolerability. As a result, European treatment guidelines have, for many years, recommended the use of topical NSAIDs as a safe and effective treatment option for OA. Following the recent approval of several topical NSAID formulations by the US Food and Drug Administration, US treatment guidelines are increasingly recommending the use of topical NSAIDs as an alternative therapy and, in some cases, as a first-line option for OA. This commentary summarizes OA treatment guidelines that are currently available and discusses their potential evolution with regard to the increased inclusion of topical NSAIDs.

## Background

Osteoarthritis (OA) is a chronic, painful, degenerative joint condition that most commonly affects the hips, knees, and hands and often requires long-term treatment to manage acute symptoms and prevent long-term complications. These complications include the destruction of articular cartilage and subchondral bone, bone remodelling, atrophy of periarticular muscles, capsular stretching, and synovitis in weight-bearing joints [1]. Additional health-related problems associated with OA include emotional stress, fatigue, and impaired sleep [2], all of which may significantly diminish the quality of a patient’s life [3]. Many patients with OA also commonly experience multiple comorbidities, such as cardiovascular, gastrointestinal, and endocrine disorders [4,5]. OA is estimated to affect nearly 27 million people in the United States [6], with annual US healthcare costs stemming from the treatment of OA estimated at $185 billion [7].

Inflammatory processes have been shown to promote disease progression in OA [8]. Consequently, oral nonsteroidal anti-inflammatory drugs (NSAIDs) have become integral to the management of OA [9-12]. However, the use of these agents has been linked to an increase in the risk of gastrointestinal [13,14], cardiovascular [15,16], and renal [17,18] adverse events (AEs). The safety of oral NSAIDs is further compromised with increasing patient age and by the use of higher doses, extended periods of use, the presence of comorbid medical conditions, and the coadministration of certain medications [14,16,19]. Selective cyclo-oxygenase-2 (COX-2) inhibitors were developed because they were thought to be safe alternatives for patients at a greater risk for peptic ulcer disease or gastrointestinal bleeding; however, population-based analyses have called their safety into question [20]. Because COX-2 has substantial expression in cardiovascular and renal tissue, concerns have been raised regarding potential adverse cardiovascular and renal effects associated with the use of these agents [21-24].

Attempts to address these issues have led to the development of new formulations with improved risk-benefit profiles in relation to earlier generations of NSAIDs. As a result, a number of NSAIDs are available worldwide as topical formulations (e.g., ibuprofen, diclofenac, and ketoprofen). Topical NSAIDs have been shown to provide analgesia through the same mechanism of action as oral NSAIDs, but because the activity of topical NSAIDs is effectively confined to the application site, systemic exposure—and consequently, the risk for gastrointestinal, cardiovascular, and renal toxicity—has been shown to be much lower than that observed with oral NSAIDs [25-31]. A Cochrane Database review of 34 studies has demonstrated that topical NSAIDs, in particular those containing diclofenac, produced fewer systemic AEs while maintaining similar effectiveness for treating chronic musculoskeletal conditions as compared to oral formulations [32].

This commentary discusses clinical guidelines for the treatment of OA in the context of the increasing availability and acceptance of topical NSAIDs in the management of OA.

### Clinical guidelines for osteoarthritis management

Currently available US guidelines for OA management include those developed by the American Academy of Orthopaedic Surgeons (AAOS), the American College of Rheumatology (ACR), the American Geriatrics Society (AGS), the American Pain Society (APS), and the Osteoarthritis Research Society International (OARSI) [9,12,33-35]. In Europe, the European League Against Rheumatism (EULAR) and the United Kingdom’s National Institute for Health and Clinical Excellence (NICE) have developed OA management guidelines [10,11,36]. Collectively, these guidelines reflect the experience of physicians across a variety of medical disciplines. However, they differ in their scope and are reliant on the therapeutic options that were available at the time they were developed.

Although the groups that formulate these guidelines generally use the same data sources (i.e., evidence-based research, expert opinion, patient experience, and cost-effectiveness analysis), different factors may be considered by each organization when developing its treatment recommendations. For instance, the AAOS and AGS guidelines reflect the perspective of specialists in orthopaedic surgery, geriatrics, and pain management, while the EULAR and OARSI guidelines primarily emphasize the findings of experts in rheumatology. The NICE guidelines are developed jointly by physicians and other healthcare professionals working in conjunction with a range of clinical researchers. As a result, the guidelines vary in their emphasis on certain treatment options, and they may even contradict each other [37]. The scope of these guidelines tends to vary as well, with some guidelines (e.g., AAOS, ACR, EULAR, and OARSI) addressing specific types of OA (i.e., knee, hip, or hand) and others (e.g., AGS, APS, and NICE) focusing on OA more generally.

Table 1 provides a summary of current guidelines regarding the use of topical analgesics in the treatment of OA [9-12,33-36].

**Table 1:** Topical analgesics in osteoarthritis (OA) guidelines

**Guideline**	**Recommendation**
American Association of Orthopaedic Surgeons (AAOS) 2013 [33]	• *Knee OA*: Strongly recommend oral or topical NSAIDs or tramadol for the pharmacologic management of patients with symptomatic OA of the knee
American College of Rheumatology (ACR) 2012 [9]	• *Hand OA*: Initial management of hand OA should include one
	• or more of the following:
	o topical capsaicin
	o topical NSAIDs, including trolamine salicylate
	o oral NSAIDs, including COX-2 inhibitors
	o tramadol
	• *Knee OA*: Initial management of knee OA should include one
	• of the following:
	o acetaminophen
	o oral NSAIDs
	o topical NSAIDs
	o tramadol
	o intra-articular corticosteroid injections
	• Topical rather than oral NSAIDs should be used in patients with hand or knee OA aged ≥75 years
American Geriatric Society (AGS) 2009 [35]	• *Localized, non-neuropathic persistent pain*: Patients with localized, non-neuropathic persistent pain may be candidates for topical NSAIDs
American Pain Society (APS) 2002 [34]	• Guidelines were published prior to FDA approval of topical NSAIDs
European League Against Rheumatism (EULAR) 2003, 2007 [10,36]	• *Hand OA*: Topical treatments are recommended over systemic treatments, especially for mild to moderate pain and when only a few joints are affected
	• *Hand or Knee OA*: Topical NSAIDs and capsaicin have clinical efficacy and are safe in the treatment of hand or knee OA
National Institute for Health and Clinical Excellence (NICE, United Kingdom) 2008 [11]	• *Hand or Knee OA*:
	o Topical NSAIDs should be considered for pain relief in addition to nonpharmacologic treatment
	o Topical NSAIDs and/or acetaminophen should be considered ahead of oral NSAIDs, COX-2 inhibitors, or opioids
Osteoarthritis Research Society International (OARSI) 2008 [12]	• *Knee OA*: Topical NSAIDs and capsaicin may be effective as adjunctives and alternatives to oral analgesics/anti-inflammatory agents in patients with knee OA

AAOS = American Association of Orthopaedic Surgeons; ACR = American College of Rheumatology; AGS = American Geriatric Society; APS = American Pain Society; COX = cyclo-oxygenase; EULAR = European League Against Rheumatism; FDA = US Food and Drug Administration; GI = gastrointestinal; NICE = National Institute for Health and Clinical Excellence; NSAID = nonsteroidal anti-inflammatory drug; OA = osteoarthritis; OARSI = Osteoarthritis Research Society International.

Adapted from Hochberg MC et al. *Arthritis Care Res (Hoboken)* 2012, **64**:465-474; Jordan KM et al. *Ann Rheum Dis* 2003, **62**:1145-1155; National Collaborating Centre for Chronic Conditions. *Osteoarthritis: National Clinical Guideline for Care and Management in Adults.* London: Royal College of Physicians; 2008; Zhang W et al. *Osteoarthritis Cartilage* 2008, **16**:137-162; *Treatment of Osteoarthritis of the Knee Evidence-Based Guideline 2nd Edition*. Rosemont, IL: American Academy of Orthopaedic Surgeons; May 2013; Simon LS et al. *Guideline for the Management of Pain in Osteoarthritis, Rheumatoid Arthritis, and Juvenile Chronic Arthritis, 2nd edition.* Clinical Practice Guidelines no. 2. Glenview, IL: American Pain Society; 2002; American Geriatrics Society Panel on the Pharmacological Management of Persistent Pain in Older Persons. *J Am Geriatr Soc* 2009, **57**:1331-1346; Zhang W et al. *Ann Rheum Dis* 2007, **66**:377-388.]

The NICE guidelines are the first and only to recommend that all patients receive first-line treatment with a topical NSAID before using an oral NSAID [11]. In the NICE guidelines, oral NSAIDs, selective COX-2 inhibitors, and opioids are considered to be adjunctive or second-line therapies [11]. The positioning of topical NSAIDs in the NICE guidelines reflects the extensive experience that physicians in Europe have with topical preparations, as well as several randomized, controlled trials conducted with a topical NSAID and placebo, with or without an oral comparator arm. The results of these studies have demonstrated that topical NSAIDs improve symptom severity, daily functioning, and quality of life in adults with OA to a greater degree than placebo, with results comparable to the efficacy of oral formulations [38-40]. In addition, NICE has recently completed the Public Consultation Phase for developing new guidelines, so an updated version of these guidelines is expected to be published soon.

The EULAR guidelines for the treatment of hand OA recommend topical treatments over systemic treatments [36]. The EULAR (knee OA) guidelines indicate that topical NSAIDs are safe and effective but provide no specific recommendations regarding their role in the management of OA [10,35]. OARSI recommends topical NSAIDs only as second-line or adjunctive therapy in patients whose pain does not respond to treatment with acetaminophen [12].

Though widely used in Europe, topical NSAIDs have only recently become available in the United States for the treatment of OA. The first topical NSAIDs approved by the US Food and Drug Administration (FDA) were diclofenac 1% sodium gel, approved in 2007, and diclofenac sodium 1.5% topical solution in 45.5% dimethyl sulfoxide (DMSO), approved in 2009. Topical NSAIDs are included in each of the clinical guidelines for the treatment of OA, with the exception of those put forth by the APS, which were published in 2002 and thus preceded the availability of topical NSAIDs in the United States [34]. Similar to EULAR, the AGS guidelines provide no specific recommendations regarding the role of topical NSAIDs in the management of OA [10,35].

The technical expert panel of the ACR concluded in their recently updated guidelines that topical NSAIDs should be considered among several first-line therapies for the treatment of hand or knee OA. In addition, the ACR specifically recommends the use of topical rather than oral NSAIDs for patients with hand or knee OA who are ≥75 years of age [9]. Presumably, this age cutoff was chosen to lower the risk for developing complications related to the use of oral NSAIDs in an older population, but the ACR guidelines do not specifically describe the rationale for making this recommendation.

The AAOS was the first US organization to specifically recommend topical NSAIDs as a first-line treatment option in OA. The organization makes a strong recommendation to use topical or oral NSAIDs or tramadol as first-line pharmacologic treatment options for the management of OA of the knee [33]. Interestingly, these guidelines determined that the data available for acetaminophen, opioids, and pain patches was inconclusive regarding their efficacy for treating OA of the knee.

### Recommendations for future research and guideline development

As the amount of data available in the literature supporting the efficacy and safety of topical NSAIDs continues to grow, treatment guidelines can be expected to evolve as well. Several double-blind, randomized clinical trials have been published that support the recent shift in US-based guidelines to include the FDA-approved topical NSAIDs as first-line agents.

In two of these studies, patients with OA were treated with topical diclofenac sodium 1% gel. One study was 8 weeks in duration and enrolled patients with OA of the hand, while the other, a 12-week study, enrolled patients with OA of the knee. In both of these studies, topical diclofenac sodium 1% gel produced significant improvements in OA pain intensity and functioning relative to placebo as early as the end of treatment week 1, with continued improvement over the course of the studies [41].

In another study, patients with OA of the knee were treated using diclofenac sodium 1.5% topical solution in 45.5% DMSO for 12 weeks. Topical diclofenac produced significant improvement compared with placebo on measures of pain and physical functioning, as well as patient global assessment. The topical diclofenac arm of this study produced a treatment effect that was comparable to slow-release oral diclofenac [42].

Recent analyses of short-term clinical trials conducted with topical NSAIDs suggest that this class of treatment may be a preferred monotherapy option for patients with OA in single or multiple superficial joints, particularly among those who are at an increased risk for NSAID-related systemic AEs [25-28]. In a longer-term, 12-month, post hoc analysis conducted in patients with OA of the knee stratified by age (ie, <65 or ≥65 years) and treated with diclofenac sodium 1% gel, the overall rates of gastrointestinal and cardiovascular AEs were generally similar for both age groups [43]. The true safety profile of topical NSAIDs can only be determined after many years of use in a large patient population. Therefore, long-term, head-to-head trials comparing the efficacy and safety of topical versus oral NSAIDs may be necessary. Future research should also include patients with OA involving multiple joints and comorbid conditions to confirm the safety of topical NSAIDs in patients who may have the highest risk for developing tolerability issues.

Clinical guidelines on the use of topical NSAIDs as safe and effective alternatives to oral NSAIDs should be regularly evaluated as new OA research emerges. Many current treatment guidelines for OA already suggest minimizing NSAID exposure and the risk of complications by prescribing the lowest effective oral dose for the shortest duration of time, but initiating treatment with topical NSAIDs, as recommended in the NICE guidelines, may help to even more effectively mitigate such risks. If long-term tolerability data demonstrate that there is a significant reduction in the frequency of cardiovascular and gastrointestinal AEs with topical NSAIDs compared to oral NSAIDs, revising or removing the black box warning requirement for topical NSAIDs also may be warranted.

## Conclusions

Currently, only the NICE and AAOS recommend topical NSAIDs as first-line treatment for OA of the knee. Additionally, EULAR guidelines recommend these agents as a first-line option for OA of the hand. Other guidelines, such as ACR, recommend topical NSAIDs as first-line treatment only for select, high-risk patient populations. However, several US guidelines have not been updated since the recent FDA approval of topical diclofenac formulations. Reevaluation of those guidelines on the basis of emerging research should help to streamline OA management recommendations and further improve patient outcomes in the safest possible manner. Recommendations for the management of OA will likely continue to evolve to include the increased and earlier use of topical NSAIDs.

## Abbreviations

AAOS: American Association of Orthopaedic Surgeons; ACR: American College of Rheumatology; AE: Adverse event; AGS: American Geriatric Society; APS: American Pain Society; COX: Cyclo-oxygenase; DMSO: Dimethyl sulfoxide; EULAR: European League Against Rheumatism; FDA: US Food and Drug Administration; NICE: National Institute for Health and Clinical Excellence; NSAID: Nonsteroidal anti-inflammatory drug; OA: Osteoarthritis; OARSI: Osteoarthritis Research Society International; US: United States.

## Competing interests

The author declares no competing interests.

## Author contribution

CB provided substantial intellectual contributions at all stages of the preparation of this manuscript and approved the final manuscript.

## Pre-publication history

The pre-publication history for this paper can be accessed here:

http://www.biomedcentral.com/1471-2474/15/27/prepub
